# Social Network Structure Is Related to Functional Improvement From Home-Based Telerehabilitation After Stroke

**DOI:** 10.3389/fneur.2021.603767

**Published:** 2021-02-02

**Authors:** Archana Podury, Sophia M. Raefsky, Lucy Dodakian, Liam McCafferty, Vu Le, Alison McKenzie, Jill See, Robert J. Zhou, Thalia Nguyen, Benjamin Vanderschelden, Gene Wong, Laila Nazarzai, Jutta Heckhausen, Steven C. Cramer, Amar Dhand

**Affiliations:** ^1^Harvard Medical School, Boston, MA, United States; ^2^Department of Neurology, Brigham and Women's Hospital, Boston, MA, United States; ^3^Department of Neurology, University of California, Irvine, Irvine, CA, United States; ^4^Department of Physical Therapy, Chapman University, Orange, CA, United States; ^5^Department of Psychological Science, University of California, Irvine, Irvine, CA, United States; ^6^Department of Neurology, University of California, Los Angeles, Los Angeles, CA, United States; ^7^California Rehabilitation Institute, Los Angeles, CA, United States; ^8^Network Science Institute, Northeastern University, Boston, MA, United States

**Keywords:** stroke, telerehabilitation, social networks, stroke recovery, telemedicine

## Abstract

**Objective:** Telerehabilitation (TR) is now, in the context of COVID-19, more clinically relevant than ever as a major source of outpatient care. The social network of a patient is a critical yet understudied factor in the success of TR that may influence both engagement in therapy programs and post-stroke outcomes. We designed a 12-week home-based TR program for stroke patients and evaluated which social factors might be related to motor gains and reduced depressive symptoms.

**Methods:** Stroke patients (*n* = 13) with arm motor deficits underwent supervised home-based TR for 12 weeks with routine assessments of motor function and mood. At the 6-week midpoint, we mapped each patient's personal social network and evaluated relationships between social network metrics and functional improvements from TR. Finally, we compared social networks of TR patients with a historical cohort of 176 stroke patients who did not receive any TR to identify social network differences.

**Results:** Both network size and network density were related to walk time improvement (*p* = 0.025; *p* = 0.003). Social network density was related to arm motor gains (*p* = 0.003). Social network size was related to reduced depressive symptoms (*p* = 0.015). TR patient networks were larger (*p* = 0.012) and less dense (*p* = 0.046) than historical stroke control networks.

**Conclusions:** Social network structure is positively related to improvement in motor status and mood from TR. TR patients had larger and more open social networks than stroke patients who did not receive TR. Understanding how social networks intersect with TR outcomes is crucial to maximize effects of virtual rehabilitation.

## Introduction

Stroke is the leading cause of acquired adult disability worldwide (1). Of the 15 million people worldwide who suffer from a stroke each year, 5 million are permanently disabled and require extensive post-stroke care. Rehabilitation, which typically includes physical, occupational, and speech therapy, can significantly improve outcomes (2). It is crucial that patients have access to rehabilitation during the first 3 months of recovery, as up to 80% of preventable stroke readmissions can be linked to health habits and post-stroke care (3, 4). However, the engagement and compliance with high-dose rehabilitation is variable and deficient in many patients with stroke (5, 6).

The coronavirus disease 2019 (COVID-19) pandemic has strained our ability to deliver critical post-stroke care. Efforts to curb COVID-19 spread have left many patients isolated from medical services they may otherwise access. These changes have especially impacted patients with stroke, many of whom are over 65 years of age and at high risk for serious illness due to COVID-19. Several countries report a decline in acute stroke admissions anywhere from 50 to 80% (7). Access to outpatient rehabilitation clinics has become significantly limited (8, 9). In addition, patients are frequently isolated from caregivers who may provide valuable support during the recovery process (10). Finally, the social isolation that many patients face during the pandemic is itself an individual risk factor for stroke recurrence (11). These barriers highlight a need for remote, accessible models of post-stroke care that account for patients' social support systems.

Telerehabilitation (TR) may address many of these limitations during the pandemic and, if integrated into routine practice, deliver valuable post-stroke care to under-resourced areas (12). In a prior study, we showed that 6 weeks of TR, targeting arm motor deficits after stroke, led to clinically significant motor gains equivalent to gains from a comparable dose of in-clinic therapy (13). We have yet to evaluate the efficacy of TR in the context of patients' social support systems, which are especially important in home-based programs that require adherence over several months. To this end, we developed a 12-week home-based TR program for stroke patients and routinely assessed motor status and mood. We then used PERSNET, a validated personal network analytic tool, to quantify the structure and composition of each patient's social network and evaluated which social factors might be important to achieve motor gains and improved mood from TR (14).

Our hypothesis, based on our prior research, was that larger network size would be associated with better stroke rehabilitation outcomes in the context of TR (15).

## Materials and Methods

### Participants

All patients were enrolled at the University of California, Irvine. Protocols were approved by the University of California, Irvine's institutional review board. Subjects were recruited from the community using advertisements and mailers. All participants provided written consent for participation with the understanding that they could withdraw from the study at any time. Participants did not receive any compensation for participation. Key inclusion criteria were: age ≥18 years; stroke onset any time prior to study entry; arm motor deficits with an arm motor Fugl Meyer score (FM-A) of 28-66 out of 66, and if >59, must also have a Box and Blocks score on the paretic side that is >25% lower than on the non-paretic side; and minimum level of arm functioning remaining with a Box and Blocks score on the paretic arm that is ≥3 blocks in 60 s. Exclusion criteria were: a major, active, coexistent neurological, or psychiatric disease; a diagnosis (apart from the index stroke) that substantially affects paretic arm function; severe depression, defined as Geriatric Depression Scale Score >11/15; significant cognitive impairment, defined as Montreal Cognitive Assessment score <22/30; and deficits in communication that interfere with reasonable study participation.

### Study Design

The overall study was a longitudinal examination of TR effects on recovery outcomes, with a planned nested cross-sectional analysis of social networks. This paper focused on the social network analysis, with the results of the overall study presented separately (16). Patients initially underwent an in-person assessment to measure baseline function. Baseline measures occurred over 2 visits and included arm motor Fugl-Meyer score (FM-A), leg motor Fugl-Meyer score (FM-L), Box and Blocks score (unaffected hand assessed before affected hand), gait velocity during 10-meter walk test, Geriatric Depression Scale, Montreal Cognitive Assessment, and Nottingham Sensory Assessment. These range of measures assessed upper and lower extremity sensorimotor status, gross motor function, cognitive function, and mood.

Following baseline assessment, a TR system was set up in each patient's home. The 12-week TR plan was created by a licensed occupational or physical therapist (OT/PT) following the live exam that took place at each patient's two baseline exams. During 6 training days a week, the patients completed 1 h of therapy that included functional games involving the upper and lower extremities (from 33 available), exercises (114 available), and 5 min of stroke education using a Jeopardy style game that focused on stroke prevention. Patients were allowed to use the TR system to play functional games after the day's assignments were completed or on rest days.

On selected days, training sessions included a videoconference with a licensed OT/PT 3 times/week during weeks 1–2, 2 times/week during weeks 3–4, and 1 time/week during weeks 5–12. In the videoconferences, therapists remotely assessed patients, recorded patient weight on a study provided scale, reviewed progress, provided feedback, and answered questions. Therapists regularly updated treatment plans based on feedback from the video conferences as well as data on system usage and game scores, which were collected in real-time as patients engaged with the system.

Live assessments were conducted at the clinic on week 6 and week 12. FM-A, FM-L, and 10-m walk time were reassessed at 6 weeks. All baseline measures were reassessed at 12 weeks. Since TR patients were enrolled with arm motor deficits, we chose 12-week improvement in FM-A, a measure of sensorimotor impairment in the upper arm, as the primary metric for motor gains. 12-week improvement in walk time, a measure of day-to-day motor function, was our secondary metric for motor gains. 12-week improvement in Geriatric Depression score was chosen to assess changes in depressive symptoms.

### Social Network Analysis

At week 6, the subjects completed the PERSNET social network survey. The PERSNET survey was an adaptation of the General Social Survey (17) and a national survey of personal networks and health (18). The research team administered the survey to the patient and recorded the patient's responses in REDCap, a web-based application for online surveys. We have demonstrated the utility of this method in health outcomes research, including studies of stroke and multiple sclerosis (19, 20).

The main sections of the survey were a name generator, name inter-relater, and name interpreter. In the name generator section, participants named people with whom they had discussed important matters, socialized, or sought support in the last 3 months. For instance, patients were asked: “From time to time, most people discuss important personal matters with other people. Looking back over the last 3 months, who are the adults with whom you discussed an important personal matter?” Patients could then choose which names to include in their network map.

In the name inter-relater section, participants determined the connections among all persons in the network and evaluated the strength of the relationship ties. Participants were asked: “Is (NAME 1) a total stranger, especially close, or in-between with (NAME 2)?” Relationship strength was quantified as 0 for total stranger, 1 for in-between, and 2 for especially close. In the name interpreter section, participants answered questions about characteristics and health habits of each individual in the network. For instance, patients were asked: “Which people in your network do you think have exercised at least 3–4 times a week in the past 3 months?” Options for each network member were “Yes, No, or Don't know.” To avoid survey fatigue, name inter-relater and name interpreter data were collected for only the first 10 individuals named by the participant.

We recorded measures of each patient's personal network structure and composition. Network structure is a quantitative description of the ties in a patient's social network. For example, the number of network members is network size, and the number of ties divided by the total number of possible ties is density. Network composition is the proportion of characteristics across all persons in the network. For example, we calculate the percentage of persons who exercise more than three times per week. Network size includes all unique inputted names, whereas density, constraint, effective size, and maximum degree are calculated using tie information from the first 10 names. Size, density, and maximum degree are unweighted measures, in which we do not account for tie strength. Constraint and effective size are weighted measures in which we account for the proportional strength of the relationship between two network members. A definition of each network structure and composition metric is provided in Table 1.

**Table 1:** PERSNET-derived social network metrics.

**Network variable**	**Definition**	**Equation**
**Structural variables**		
Network size	Number of individuals in the network, excluding the patient	Size = *N*where *N* is the number of network members.
Network density	Number of ties divided by number of possible ties	$\text{Density}=\frac{2L}{N\left(N-1\right)}$ where *L* is the number of ties, and *N* is the number of network members.
Network constraint	The extent to which the patient is connected to network members who are connected to one another	Constraint of i′s network= ${\left(p_{ij}+\sum_{q}p_{iq}\times p_{qj}\right)}^{2}$ where *i* is the patient, *q* and *j* are network members, *p*_*ij*_ is the proportional strength of *i*'s relation with *j*, *p*_*iq*_ is the proportional strength of *i*'s relation with *q*, and *p*_*qj*_ is the proportional strength of *q*'s relation with *j*.
Effective size	Number of non-redundant members in the network	Effective size of *i*′s network = $\underset{j}{\sum }\left[1-\underset{q}{\sum }P_{iq}\times m_{jq}\right],q\neq i,j$ where *i* is the patient, *q* and *j* are network members, and $\sum_{q}p_{iq}\times m_{jq}$ measures the portion of *i*'s relationship with *j* that is redundant to *i*'s relationships with other primary contacts.
Maximum degree	Highest number of ties by a network member, excluding the patient	Maximum Degree = *L*_max_where *L*_max_ is the highest number of ties incident on a single network member.
**Compositional variables**		
Percentage who exercise	Ratio of network members who exercise (>3 times/week)	Ratio = $\frac{N_{yes}}{\left(N_{yes}+N_{no}\right)}$where *N*_*yes*_ is the number of nodes who share the characteristic and *N*_*no*_ is the number of nodes who do not.
Percentage who smoke	Ratio of network members who smoke (any smoking history)	
Percentage kin	Ratio of network members who are family	

Finally, we compared the network results from this cohort to 176 historical stroke controls who did not receive a post-stroke intervention, as described in a prior publication (21). Social networks were defined in the same manner using the PERSNET survey.

### Statistical Analysis

We performed a series of univariate analyses using Spearman rank-order correlation to measure the association between social network characteristics and changes in motor function and mood. We used Pearson correlation to measure the association between social network characteristics and the Medical Outcomes Study Social Support Survey (MOS-SSS), an established measure of social support. We then compared social network characteristics between TR and historical stroke control patients using a Wilcoxon rank-sum test. All analyses were completed in RStudio version 1.2.1335. As this was an exploratory pilot study, we did not adjust for covariates or correct for multiple comparisons.

## Results

Patients in the TR cohort (*n* = 13) had a median age of 69 (IQR = 52–65.5). They were 129 (52–486) days after stroke when they enrolled. Baseline characteristics, mood, and motor function of the cohort are described in Table 2.

**Table 2:** Baseline characteristics of the TR patient cohort.

	**Overall**
Number of enrolled patients	13
**Sex (%)**	
Female	4 (30.8)
Male	9 (69.2)
**Race (%)**	
White	9 (69.2)
Asian	3 (23.1)
Black or African American	1 (7.7)
Age [median (IQR)]	61 (52–65.5)
Years of education [median (IQR)]	14 (IQR=13.5–16)
Days post-stroke [median (IQR)]	129 (52–486) (range 37-1,682)
**Comorbidities (%)**	
Hypertension	10 (76.9)
Hypercholesterolemia	7 (53.8)
Diabetes mellitus	4 (30.8)
Atrial fibrillation	2 (15.4)
Shoulder pain present at baseline (%)	8 (61.5)
**Affected side (%)**	
Left	10 (76.9)
Right	3 (23.1)
**Handedness (%)**	
Left	3 (23.1)
Right	10 (76.9)

Overall, TR patients improved in stroke rehabilitation metrics over 12 weeks (Table 3). Specifically, median FM-A significantly improved from a baseline of 46 (42–57) to 59 (52.5–61.5) (*p* = 0.0005). Median gait velocity significantly improved from 0.94 (0.67–1.09) to 1.01 (0.83–1.21) (*p* = 0.0007). Geriatric Depression score significantly decreased over 12-weeks from 3 (1–5) to 1 (0–4) (*p* = 0.05). Details of these outcomes and other recovery markers are described separately (16).

**Table 3:** Functional improvement following TR intervention.

	**Baseline [median (IQR)]**	**After 12 weeks of treatment [median (IQR)]**	***p*^**a**^**
Arm motor Fugl-Meyer score (66 max)	46 (42–57)	59 (52.5–61.5)	***p*** **=** **0.0005**
Gait velocity (m/s)	0.94 (0.67–1.09)	1.01 (0.83–1.21)	***p*** **=** **0.0007**
Geriatric depression scale score (15 max)	3 (1–5)	1 (0–4)	***p*** **=** **0.05**

a *p-values using Wilcoxon signed rank-test. Bolded values are statistically significant*.

**Figure 1:**
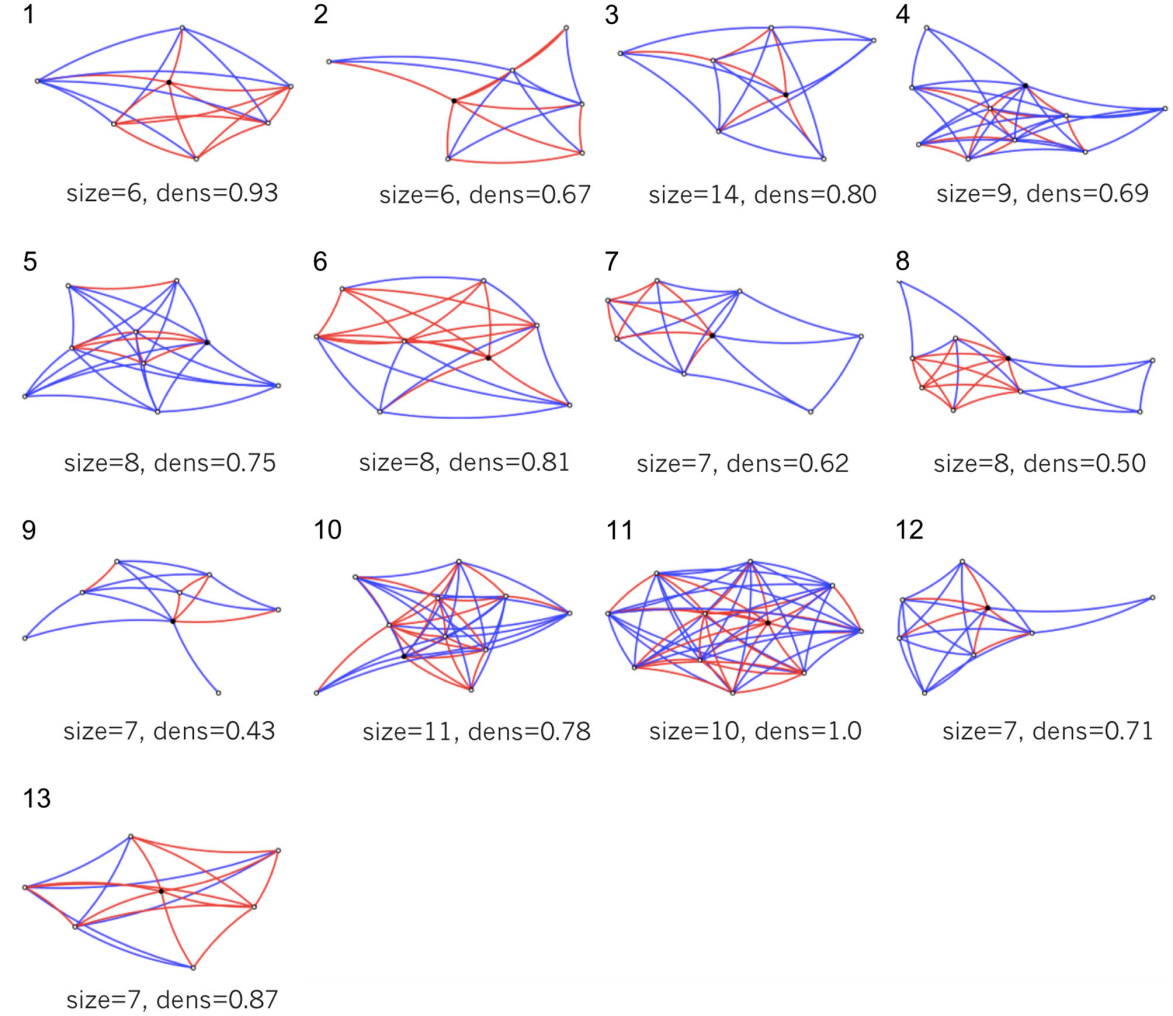
Six-week social networks of TR patients mapped with the PERSNET algorithm. The index patient is represented by a black dot, and each of the network members is represented by an open dot. Red lines represent strong ties and blue lines represent weak ties. Graphs show only the first 10 names patients chose to include in their network maps. Patient networks reveal a broad range of social structures, from large and close-knit to moderately sparse with peripheral support nodes.

The TR patients had a broad range of social networks, as shown in Figure 1. There are varying types of networks that are useful for understanding patients' social realities and planning rehabilitation. There are patients with large networks (e.g., ID10 and ID11) vs. patients with small networks (e.g., ID1 and ID2). Within the large networks of ID10 and ID11, there is a difference in density. ID10 has a star-like structure of connections usually better for informational support (e.g., hearing new ideas). ID11 has a close-knit structure usually better for instrumental support (e.g., getting a ride). The smaller networks tend to be more at-risk for reduced engagement in rehabilitation. For example, both ID1 and ID2 may require more support from external sources. ID8, ID9, and ID12 represent another pattern of network structure. Their networks have peripheral network members who are not connected to other network members. This usually occurs when the patient has made relationships in a context separate from their core relationships, such as at church or school.

In all TR patients, the mean network size was 8.3 (SD = 1.4), and mean network density was 0.74 (SD = 0.10). The mean network constraint was 0.46 (SD = 0.03), mean effective size was 3.8 (SD = 0.5), and mean maximum degree was 6.2 (SD = 1.0). In terms of composition metrics, the percentage kin was 0.49 (SD = 0.08), mean percentage who exercise was 0.51 (SD = 0.15), and mean percentage who smoke was 0.11 (SD = 0.12).

Network metrics were positively associated with improvement in stroke rehabilitation metrics. 12-week improvement in FM-A, our primary measure of motor status, was positively correlated with social network density (*r* = 0.75, *p* = 0.003). Improvement in walk time, our secondary measure of motor function, was positively correlated with social network size (*r* = 0.61, *p* = 0.025) and density (*r* = 0.080, *p* = 0.003). Additionally, 12-week improvement in Geriatric Depression score was positively correlated with network size (*r* = 0.679, *p* = 0.015; Table 4). Network size and density were not correlated within the TR cohort (*r* = 0.24, *p* = 0.42). As a validation of the social network metrics, we found that TR patients' 6-week social network size was correlated with the MOS-SSS (*r* = 0.71, *p* = 0.015), an established measure of social support. No significant correlations were observed between network size and FM-A improvement or network density and improvement in Geriatric Depression score. Network composition metrics (e.g., percentage kin) also showed no significant correlation with functional motor or mood improvement.

**Table 4:** Relationships between social network metrics and improvement in stroke rehabilitation scores.

**Social Network Metric**	**Stroke rehabilitation metric**			
		**12-week Arm motor Fugl-Meyer score improvement**	**12-week Walk time improvement**	**12-week Geriatric depression score improvement**
	Network size	*r* = 0.06	*r* = 0.62	*r* = 0.68
		*p* = 0.85	***p*** **=** **0.02**	***p=*** **0.02**
	Network density	r = 0.75	*r* = 0.78	*r* = 0.27
		***p=*** **0.003**	***p=*** **0.003**	*p* = 0.39
	Network constraint	*r* = 0.03	*r* = −0.14	*r* = 0.22
		*p* = 0.92	*p* = 0.66	*p* = 0.50
	Effective size	*r* = −0.57	*r* = −0.46	*r* = 0.06
		***p*** **=** **0.04**	*p* = 0.11	*p* = 0.86
	Maximum degree	*r* = 0.19	*r* = 0.58	*r* = −0.42
		*p* = 0.53	***p*** **=** **0.04**	*p* = 0.18
	Percentage kin	*r* = 0.17	*r* = 0.05	*r* = 0.31
		*p* = 0.57	*p* = 0.87	*p* = 0.32
	Percentage who exercise	*r* = −0.42	*r* = 0.03	*r* = −0.33
		*p* = 0.15	*p* = 0.91	*p* = 0.30
	Percentage who smoke	*r* = 0.14	*r* = 0.01	*r* = −0.06
		*p* = 0.64	*p* = 0.97	*p* = 0.85

*p-values using Spearman rank-order correlation. Bolded values are statistically significant*.

TR patients had larger and less dense networks compared to the networks of a historical control population of patients with stroke (*n* = 172). The control patients had a median age of 62.5 (IQR = 51–74). They had a median of 14 years of education (IQR = 12–16). 114 (66.3%) were White, 53 (30.8%) were Black or African American, 1 was Asian (0.6%), 2 were other (1.2%), and 2 were unknown or not reported (1.2%). Control patients' social networks were recorded 6 months after stroke, and full results are reported separately. Control patient networks had a mean network size of 6.7 (SD = 0.7) compared to a mean of 8.3 (SD = 1.4) in the TR cohort (*p* = 0.01). Control networks were denser than TR networks, with a mean density of 0.82 (SD = 0.05) compared to 0.74 (SD = 0.10) in TR patients (*p* = 0.046; Table 5). There were no significant differences in the composition, including health habits, of network members within the TR and control patient groups.

**Table 5:** Comparison of TR and control patient social networks.

	**TR cohort (*n* = 13) [mean (±SD)]**	**Control cohort (*n* = 176) [mean (±SD)]**	***p*^**a**^**
Network size	8.3 (7.0–9.7)	6.7 (6.0–7.4)	**0.01**
Network Density	0.74 (0.64–0.83)	0.82 (0.78–0.85)	**0.05**
Network Constraint	0.46 (0.42–0.49)	0.58 (0.54–0.61)	0.07
Effective size	3.8 (3.4–4.3)	2.9 (2.6–3.2)	**0.005**
Maximum degree	6.2 (5.2–7.1)	4.8 (4.3–5.2)	**0.04**
Percentage who exercise	0.51 (0.35–0.67)	0.44 (0.38–0.49)	0.41
Percentage who smoke	0.11 (−0.06–0.27)	0.17 (0.13–0.21)	0.15
Percentage kin	0.49 (0.41–0.57)	0.61 (0.56–0.67)	0.10

a *p-values using Wilcoxon signed rank-test. Bolded values are statistically significant*.

## Discussion

We evaluated the association of social network characteristics and stroke rehabilitation outcomes following 12 weeks of home-based TR. In this pilot study with limited sample size, we found that social network structural metrics were associated with changes in motor status and mood during TR. Patients with larger social networks showed greater functional improvement in walk time and reported greater decline in depressive symptoms following TR. Patients with denser, or more closely-knit, networks saw greater improvements in upper arm sensorimotor function and walk time. Finally, the TR cohort had larger and more open network compared to a control cohort of stroke patients who were not exposed to interventions, which suggests a relationship between network structure and participation in TR.

There are potential mechanisms in the literature as to how TR may provide social benefits for patients (22, 23). In a qualitative study of a 6-week TR program, patients reported improvements in their social and emotional well-being following the program (24). Several subjects noted that regular calls with therapists helped them feel less isolated. Thematic analysis also showed that social support and the perception of physical improvement influenced usage behavior of TR. Many patients shared that support from family members at home was a major motivator to continue with rehabilitation. These results suggest a reinforcing loop between social support and TR usage. Our work contributes to this literature by adding a quantitative study of individuals' social networks during TR and their relationship to post-stroke outcomes.

Conversely, social support might benefit TR, consistent with the growing evidence on the role of social networks in stroke recovery. Patients often experience a contraction of social networks following stroke as they may lose contact with friends, attend fewer group events, and avoid social activities (25). This network contraction can worsen disability, as social isolation has been associated with poorer post-stroke physical outcomes and increased risk of depression 12 months following stroke. Conversely, high levels of social support are associated with faster and more extensive recovery of functional status after stroke (26). The role of social networks in recovery must be incorporated into post-stroke rehabilitation programs in order to address the compounding effects of engagement and social isolation on outcomes.

The strength of this study is that it examines the social networks of patients as a novel cofactor in their response to a novel TR intervention. The unique design of the TR program integrates physical exercises, education, and longitudinal interaction with a therapist. Additionally, the use of a retrospective control group allows for comparisons of network structure and composition between patients with stroke who did and did not undergo TR intervention. In the broader context of telemedicine and the COVID-19 pandemic, this study offers clinically relevant insights into care delivery, social support, and patient outcomes.

There are limitations to this study. The PERSNET survey requires cognitive and linguistic capabilities which may have influenced the inclusion of patients in the TR program. The different sizes of the TR and control group cohorts may have influenced estimates of social metrics within each group. Due to the limited sample size, we could not use multivariable regression to measure the effect of confounding variables in each observed relationship. Also, due to the exploratory nature of this study, we did not correct for multiple comparisons. It would be useful to repeat this study with a larger cohort of patients with diverse stroke severity to identify confounders and understand the range of network structures with which the observed relationships may hold. Moreover, our analyses rely on a single snapshot of each patient's social network over longitudinal change.

To begin to parse social processes influencing TR performance, baseline, 6- and 12-week PERSNET assessments could capture how these networks may change over the course of TR. These additional time points would allow for greater understanding of directionality of the effects. Supplementation with standardized doses of social interaction during the initial weeks of the study could help us better understand causal relationships between network structure, social interaction, and functional improvements from TR. Finally, future interventions could include approaches to increase social interactions for TR patients with smaller or sparse social networks. TR interventions could incorporate social games where patients engage with each other or a therapist. TR may also involve collaborative exercises that require patients to reach out to weak ties and form new social connections.

In summary, we observe preliminary relationships between network structure, motor gains, and mood improvement during TR. Our analyses suggest that the health of patients' social networks may be an important factor in their interaction with home-based TR systems. During the COVID-19 crisis, and as we transition toward virtual models of care, it will be important to study interactions among telerehabilitation design, patient adherence, and social support to improve outcomes from remotely delivered therapies.

## Data Availability Statement

The original contributions generated in the study are included in the article/supplementary material, further inquiries can be directed to the corresponding author.

## Ethics Statement

The studies involving human participants were reviewed and approved by Institutional Review Board (IRB) of the University of California, Irvine. The patients/participants provided their written informed consent to participate in this study.

## Author Contributions

SC, AD, AP, and SR contributed to concept design, data collection, and writing of the manuscript. All authors participated in data analysis and revision.

## Conflict of Interest

SC is a consultant for Constant Therapeutics, MicroTransponder, Neurolutions, SanBio, Stemedica, Fujifilm Toyama Chemical Co., Medtronic, and TRCare. AD is an expert witness for Neuro Consults, LLC. LD, VL, and JS are consultants for TRCare. The remaining authors declare that the research was conducted in the absence of any commercial or financial relationships that could be construed as a potential conflict of interest.

## Abbreviations

TR: telerehabilitation
FM-A: arm motor Fugl-Meyer score
FM-L: leg motor Fugl-Meyer score.
